# The prevalence and phenotype in Brazilian patients with inflammatory bowel disease

**DOI:** 10.1186/s12876-018-0822-y

**Published:** 2018-06-18

**Authors:** Adalberta Lima Martins, Rhaisa Almeida Volpato, Maria da Penha Zago-Gomes

**Affiliations:** 1 State Management of Pharmaceutical Assistance and Ambulatory of Inflammatory Bowel Disease of the Health Department of Espírito Santo, Núcleo Regional de Especialidades de Vitória, BR 262, Km 0, Ed. Eng. Cristiano Tavares, 2° andar, Jardim América, Cariacica, Espírito Santo 29140-126 Brazil; 2 Laboratory of Estatistic-Departament of de Federal University of Espírito Santo, Av. Fernando Ferrari s/n, Goiabeiras, Vitória, Espírito Santo 29060-900 Brazil; 3 Departament of Medicine, Federal University and University Hospital Cassiano Antonio Moraes of Espírito Santo, Av. Marechal Campos, 1468, Vitória, ES 29042-755 Brazil; 4 Desembargador Ferreira Coelho 330/315 Praia do Suá, Ed. Eldorado, Vitoria, 29052210 Brazil

**Keywords:** Prevalence, Inflammatory bowel disease, Phenotype, Ulcerative colitis, Crohn’s disease, Brazil

## Abstract

**Background:**

The epidemiology of inflammatory bowel disease (IBD) varies in different countries. This study aimed to assess phenotype, medications, prevalence and incidence of IBD in the State of Espírito Santo, Brazil.

**Methods:**

Patients with IBD who utilized the Public Medication-Dispensing System of the Department of Health of Espírito Santo, between August 2012 and July 2014. Of 1484 active patients, 1048 were analyzed, which included patients of all ages.

**Results:**

The prevalence of IBD was 38.2 per 100,000 inhabitants, with ulcerative colitis (UC) being 24.1 per 100,000 and Crohn’s disease (CD), 14.1 per 100,000. The incidence of IBD was 7.7 per 100,000 inhabitants/year (UC – 5.3/100,000 inhabitants/year and CD – 2.4/100,000 inhabitants/year). Out of the 1048 patients analyzed, 669 had UC (63.84%), 357 had CD (34.06%), and 22 had unclassified inflammatory bowel disease (UIBD; 2.10%). There were 48/1048 (4.5%) pediatric patients (16 years of age or younger). On the UC phenotype (*n* = 654), we observed left-sided colitis in 247 (37.7%), pancolitis in 209 (31.9%), and proctitis in 198 patients (30.2%). Pancolitis was more frequent in pediatric patients (*p* = 0.007). CD showed a homogeneous distribution between ileitis (L1), colitis (L2), and ileocolitis (L3). Regarding the CD behavior (*n* = 352) observed the inflammatory type (B1) in 176 (50%); fistulizing (B3) in 75 (21.2%), isolated type (B3) in 29 (8.2%), and perianal fistulizing type (B3p) in 46 (13.1%). Biologic therapies were used in 154/357 (43.3%).

**Conclusion:**

The prevalence of the IBD in the state of Espírito Santo, Brazil was higher than in two other brazilian studies. There was high utilization of biologic therapies in patients with CD.

## Background

Inflammatory bowel diseases (IBD) are complex processes [1] and are associated with regional epidemiological [2–5] and phenotypical [6–8] variations. Latin America, particularly Brazil, is considered to be a region with low prevalence and incidence of IBD, though there has been an increase in incidence in Brazil [9, 10]. In 2015, Parent et al. found a prevalence of 12.8 cases/100,000 inhabitants in the northeast of Brazil [11].

According to the medication-dispensing protocol of the Ministry of Health, treatment for IBD in Brazil is provided by the federal government for all patients, including those from both the public and private systems [12–14].

**Fig. 1:**
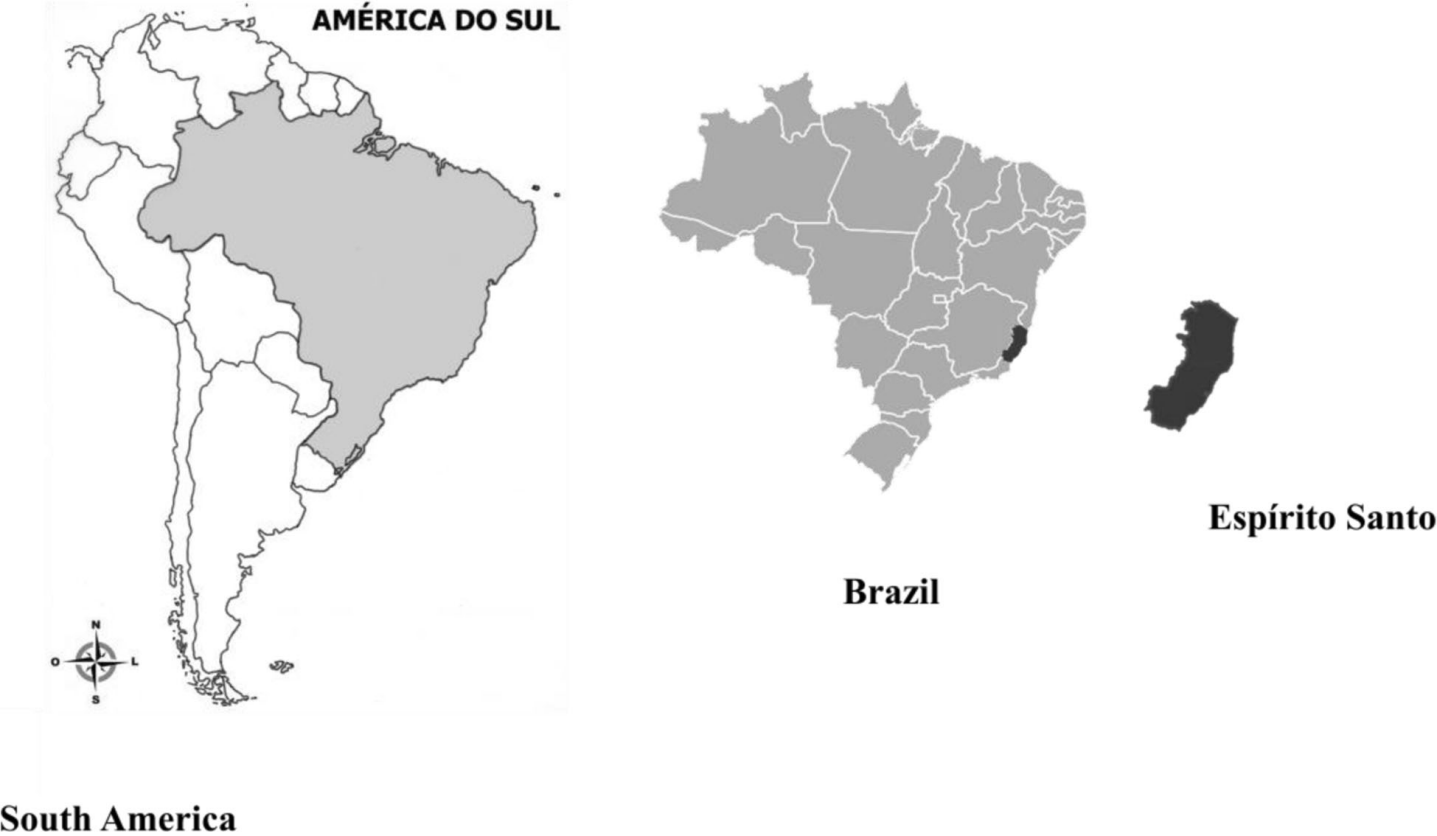
Map of Espírito Santo, Brazil, South America

Espírito Santo is a state located in the Southeast region of Brazil (Fig. 1). It has a large immigrant population of Italians and Germans [15], and in 2014, the population was 3,885,049 [16].

The purpose of this study is to evaluate the prevalence, incidence, phenotype, and medications used in patients who received treatment for IBD through the dispensing pharmacies in the State of Espírito Santo.

## Methods

### Ethical considerations

This study had used secondary data from documentation required by the Ministry of Health in order to have access to highly expensive medication. As the data is secondary and was obtained through medical reports, there was no parent’s consent form used for the patients under 16 years old. There was no identification of the study’s subjects, preserving the confidentiality and privacy of the study’s candidates, and were approved by the Ethics and Research Committee of the Nossa Senhora da Gloria Children’s Hospital (CAAE 19602813.8.0000.5069) after obtaining authorization from the State Office for Pharmaceutical Assistance.

### Study location

The study was conducted in the Citizen’s State Pharmacy sector of the Espírito Santo Office for Pharmaceutical Assistance, which is responsible for dispensing medications for patients with IBD in all State.

### Sample analyzed

**Fig. 2:**
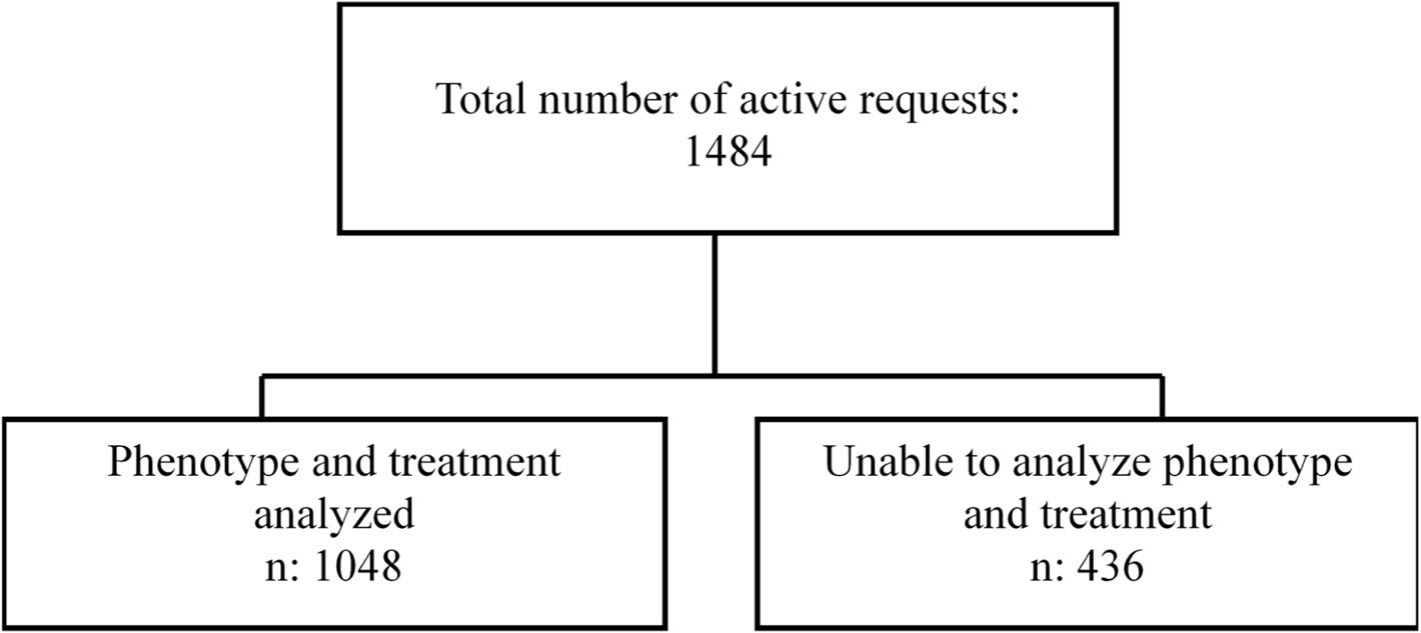
Flowchart for the analysis of medication dispensing requests for IBD in Espírito Santo, Brazil

The sample included 1484 patients, with confirmed diagnosis, of all ages with IBD in the state of Espírito Santo (Fig. 1) who received medications through the Federal Government between August 1, 2012 and July 31, 2014 and for whom the incidence and prevalence of IBD was determined. Phenotype and treatment information was not available for 436 patients, and therefore data for 1048 patients were used (Fig. 2).

### Study design

In medication-dispensing services the evaluation is conducted by only one gastroenterologist physician, in this case, the main author, who was responsible for dispensing the medication for IBD, and data analyzed were obtained through the analysis of administrative requests of these medications, and included medical report, endoscopy exams, histopathology and imaging tests, which followed the Clinical Protocols and Therapeutic Guidelines of the Brazilian Government [13, 14]. The diagnosis of IBD and the phenotype were established according to the Montreal criteria [17] for Crohn’s disease (CD), ulcerative colitis (UC). The patients whose endoscopic examination, image and histopathological examination, laboratorial, associated to medical reports describing the difficulty to define CD and UC the terminology unclassified inflammatory bowel disease (UIBD) was applied.

Dependent variables included the diagnosis, IBD classification, new cases (patients who received the diagnosis within one year of starting the administrative request), chronic cases (patients who received the diagnosis more than one year from starting the administrative request), and medications. Independent variables included age and gender.

### Study limitations

The study was conducted with secondary data and some information has been compromised, such as in some cases the endoscopic report was not included and only the histopathology report was analyzed. Not all CD patients included in the study had an upper gastrointestinal endoscopy/biopsy, and magnetic resonance and some older documents have been damaged due to time, making it impossible to define the localization of the disease in some cases.

In Brazil, medications for IBD are expensive and provided by the Public Health System for patients treated in the public and private systems. However, it is possible that some patients in the private system obtain their medications directly from drugstores without utilizing the public system.

### Statistical analysis

An Excel spreadsheet was used to collect the data, and the data were analyzed using *SPSS Statistics 20.0* software. Data were tabulated and analyzed through descriptive analysis of frequencies, percentages, averages, and standard deviations (SD). To determine associations between categorical variables, achi-square test was used, and Fisher’s exact test was also used when appropriate. Student’s t-test or ANOVA were used for quantitative variables. A *p* value of < 0.05 was considered statistically significant.

Data from the Brazilian Institute of Geography and Statistics was used to calculate prevalence and incidence based on the last census in 2014, in which the population of Espírito Santo was 3,885,049 [16]. To calculate prevalence, the total number of active requests at the Citizen’s State Pharmacy (1484) was used. To calculate incidence, new cases arising in the second year of the study were used (August 1, 2013 to July 31, 2014).

## Results

There were 1484 registered patients who received medication in the state of Espírito Santo. Considering the population in the region in 2014, the prevalence of IBD in the state was 38.2/10^5^ inhabitants. From these, 935 (63%) were diagnosed with UC and 549 (37%) were diagnosed with CD, yielding prevalence of 24.1 per 100,000 and 14.1 per 100,000 inhabitants, respectively. The incidence was calculated based on new cases in 2014 (298 patients – 204 UC and 94 CD), with an incidence of IBD of 7.7/100,00 inhabitants/year (UC 5.3/100,000 inhabitants/year and CD 2.4/100,000 inhabitants/year).

1Demographic data are summarized in Table 1 and refer to the 1048 patients whose administrative requests were reviewed, of whom 669 (63.84%) had a diagnosis of UC, 357 (34.06%) had a diagnosis of CD, and 22 (2.10%) had a diagnosis of UIBD.

**Table 1:** Distribution of ulcerative colitis and Crohn’s disease

Demographic variables		IBD				
		*n* = 1026				
		UC		CD		*p*
		*n*	%	*n*	%	
Year (Yr)	2013	339	62.8%	207	37.92%	0.043
	2014	330	68.75%	150	31.25%	
Age at diagnosis (A)^a^	A1: ≤ 16	23	3.40%	25	7.00%	
	A2: 17–40	318	47.50%	208	58.30%	0.001
	A3: > 40	328	49.00%	124	34.70%	
Gender	Female	407	60.80%	195	54.60%	0.156
	Male	262	39.20%	162	45.40%	
New cases	2013	215	51.3%	117	55.7%	0.346
	2014	204	48.7%	94	44.3%	
Old Cases	2013	124	49.6%	90	61.6%	0.058
	2014	126	50.4%	56	38.4%	

^a^Age at diagnosis is not a phenotypic element of the Montreal classification for ulcerative colitis; *UC* ulcerative colitis, *CD*, Crohn’s disease

The disease phenotype could not be determined for all patients due to a lack of information in the administrative requests. The phenotypes evaluated are available in Tables 2, 3 and 4. For pediatric patients (16 years of age or younger), 48/1048 (4.5%) patients had IBD, among whom 23/48 (48%) had UC and 25/48 (52%) had CD in Table 1. Pancolitis was more frequent in pediatric patients (*p* = 0.007), available in Table 2. In Crohn’s disease the inflammatory and perianal fistulizing behavior were more frequent, Table 4.

**Table 2:** Clinical characteristics of ulcerative colitis (*n* = 654)

Phenotypic elements		Extension						
		E1		E2		E3		*p*
		Proctitis		Left-sided colitis		Pancolitis		
		n	%	n	%	n	%	
Age at diagnosis (A)^a^	A1: ≤ 16	3	13.0	6	26.1	14	60.9	0.007
	A2: 17–40	89	28.4	116	37.1	108	34.5	
	A3: > 40	106	30.5	125	39.3	87	26.8	
Gender	Female	126	31.5	153	38.2	121	30.2	0.469
	Male	72	28.3	94	37.0	88	32.0	

^a^Age at diagnosis is not a phenotypic element of the Montreal classification for ulcerative colitis

**Table 3:** Clinical characteristics of Crohn’s disease (*n* = 357)

Phenotypic elements		Total DC	
		*n*	*%*
Location (L) (*n* = 353)	L1: terminal ileum	111	31.4
	L2: colonic	102	28.9
	L3: ileocolonic	109	30.9
	L4: isolated upper disease	11	3.2
	L1 + L4	8	2.3
	L3 + L4	12	3.4
Behavior (B) (*n* = 352)	B1: nonstricturing/non-fistulizing	176	50.0
	B2: stricturing	56	15.9
	B3: fistulizing	29	8.2
	B1p^a^	27	7.7
	B2p^a^	18	5.1
	B3p^a^	46	13.1
Gender	Female	195	54.6
	Male	162	45.4

^a^*p* perianal disease modifier

**Table 4:** Crohn disease ‘s behavior in relation to age and gender

Phenotypic elements		Behavior (B)												
		B1		B2		B3		B1p		B2p		B3p		*p*
		n	%	n	%	n	%	n	%	n	%	n	%	
Age at diagnosis (A)	A1: ≤ 16	10	5.6	0	0.0	2	6.9	3	11.1	1	5.6	9	19.6	0.001
	A2: 17–40	90	50.9	30	53.6	20	69.0	20	74.1	16	88.8	29	63.0	
	A3: > 40	77	43.5	26	46.4	7	24.1	4	14.8	1	5.6	8	17.4	
Gender	Female	110	62.1	26	46.4	16	55.2	10	37.0	11	61.1	22	47.8	0.042
	Male	67	37.9	30	53.6	13	44.8	17	63.0	7	38.9	24	52.2	

*B1* nonstricturing/non-fistulizing, *B2* stricturing, *B3* fistulizing, p-perianal disease modifier

**Fig. 3:**
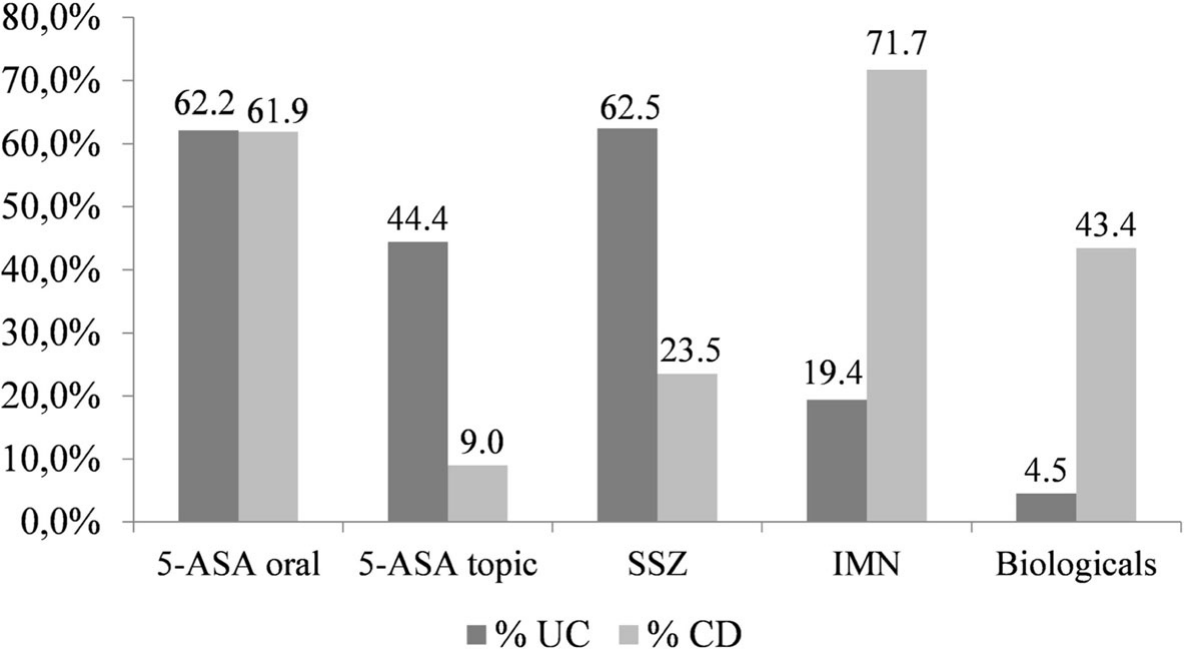
Distribution of medications used for ulcerative colitis and Crohn’s disease UC: ulcerative colitis; CD: Crohn’s disease;5-ASA: mesalazine, SSZ: sulfasalazine, IMN: immunomodulators (azathioprine and methotrexate)

Medication dispensing for patients with IBD is shown in Fig. 3. For UC patients, 591/654 (90,3%) treated with oral aminosalicylates, 438 (74%) have left-sided colitis or pancolitis and those treated with azathioprine, 110/127 (86,8%) had left-sided colitis and pancolitis. The biological treatment was prescribed for 30 (4,5%) patients with UC, with pancolitis 21 (70%) and left-sided colitis 9 (30%). For CD patients, treatment with the immunomodulator was prescribed in 253/357 (70,9%), and in 153/357 (43,4%) of the cases the biological therapy was used, due to perianal involvement 71/152 (46,7%).

## Discussion

In Brazil the prevalence of IBD in the state of Espírito Santo (38,2/100.000 inhabitants), was higher. Parente et al. in Piauí, Brazil, in 2015 [11] analyzing a University Hospital in the northeast region of Brazil found a prevalence of 12.8 cases/100,000 inhabitants and Victoria et al. in São Paulo, Brazil, between 2001 and 2005 [9], analyzing a University Hospital in the southeast region of Brazil found a prevalence of 22.6 cases/100,000 inhabitants, which only analyzed reference points (University Hospitals). The prevalence of IBD in the state of Espírito Santo, Brazil, was higher (38.2/10^5^inhabitants) because we had data from patients from the whole State, which included the public and private healthcare system. The incidence of IBD in Espírito Santo was also higher than the incidence found by Parente et al. in 2015 [11], when the institution’s incidence peaked at 1.53 cases/100,000 in 2007, which is similar to the data from Porto Rico in 2005 [10, 18]. The incidence of UC was closer to the values found by Victoria et al. in São Paulo [9] and equivalent to those in Porto Rico (Central America) [10, 18] but higher than those in other countries in Central and South America [10].

The evaluation of frequency distributions of IBD showed a predominance of UC, similar to the study published by Burisch et al. in 2014 [19], which compared Eastern and Western Europe and observed a predominance of UC in both regions. The same is observed for Brazilian studies [9, 11, 20]. The frequency distribution between the two forms of IBD shows geographic variations, with a higher predominance of UC in Nordic countries [8]. There is a tendency for CD to predominate in developed countries such as Canada and the USA [8]. In some countries in Eastern Europe, there is an equal frequency of both forms of IBD [8].

For pediatric patients (16 years of age or younger), 48/1048 (4.5%) patients had IBD, among whom 23/48 (48%) had UC and 25/48 (52%) had CD. These frequencies are lower than the overall means of 10 to 25% of pediatric patients with UC and CD, respectively, reported in the literature [21, 22]. However, the frequency was similar to that observed in a study performed in Western and Eastern Europe [19], in which 45/1560 (3%) of patients with IBD were younger than 15 years of age.

Evaluation of UC phenotype showed that left-sided colitis was more frequent, followed by pancolitis and proctitis, which occurred with relatively similar frequency. These results were similar to those found in a comparative study between Western and Eastern Europe [19], which observed left-sided colitis (E2) in 41 and 46% of patients, followed by pancolitis (E3) in 38 and 32% of patients, and proctitis (E1) in 20 and 22% of individuals from Western and Eastern Europe, respectively. These were also similar to the results of a Brazilian study by Parente et al. from 2015 [11]. The distribution and location of CD is relatively homogeneous and stable during its progression [23, 24]. In the current study, there was equivalence between the ileal (L1), colonic (L2), and ileocolonic (L3) forms, which is in agreement with data from some studies [2, 17, 24] but differs from the results of Brazilian studies [11, 20]. Regarding the involvement of the upper gastrointestinal tract (UGI) in isolation or in association with other locations, we observed a frequency of 8.8% (31/353), which is similar to the values of 10 to 15% previously reported in the literature [24].

In the current study, we observed that the inflammatory behavior was in agreement with data from the literature [11, 19, 25]. Perianal involvement was more frequent in younger patients, which characterizes a more severe presentation of the disease in this group of patients. Similar results were observed by Parente et al. [11] and in the European study by Burisch et al. in 2013 [2]. The fistulizing form occurred in 21.2% of patients, whereas it was isolated (B3) in 8.2% of patients and associated with perianal involvement (B3p) in 13.1% of patients. Our data are similar to data from Asia (19%) [3] and the study by Parente et al. [11], which found the B3 form in 13% of patients and the B3p form in 5% of patients.

Regarding the distribution of medications, we observed the use of biologic therapies in 43.3% of patients with CD, which suggests the occurrence of more severe CD; however, more studies are needed to assess the association between treatment and phenotype. Regarding the use of systemic aminosalicylates, we observed a higher frequency in patients with CD, which is not in accordance with current management guidelines, in which they are indicated for mild forms of the disease located in the ileum and for colonic forms of CD, for which there is evidence of efficacy [14, 26–29].

## Conclusion

The present study found a tendency for higher prevalence and incidence of IBD in Brazil, with values approaching those of regions with moderate incidence and prevalence [4, 8]. Our region is comprised of European immigrants, with one of the largest populations of Italians in Brazil [15, 30], which could justify the data found in our study. However, to date, there have been no studies to assess factors related to etiopathogenesis. Regarding the distribution of demographic and phenotypic data, there were no large differences compared to studies from other regions. Understanding of the epidemiology and phenotypic characteristics can contribute to a better organization and political-economic planning of public health.

## Abbreviations

5-ASA: Mesalazine
CAAE: Certificate of Presentation for Ethical Consideration
CD: Crohn’s disease
IBD: Inflammatory bowel disease
IMN: Immunomodulators
SSZ: Sulfasalazine
UC: Ulcerative colitis
UIBD: Unclassified inflammatory bowel disease
